# Editorial: Insights in obstetrics and gynecology: 2023

**DOI:** 10.3389/fmed.2025.1599095

**Published:** 2025-05-06

**Authors:** Sarah M. Cohen, Simcha Yagel

**Affiliations:** Obstetrics and Gynecology, Hadassah Medical Center, Jerusalem, Israel

**Keywords:** reproductive medicine, assisted reproduction (ART), obstetrics, obstetric complications, preterm birth, gynecologic tumor management, patient-centered care

This report presents a collection of 19 publications, including original research, systematic review, short communications, and case reports from the Research Topic “*Insights in Obstetrics and Gynecology: 2023*,” published in *Frontiers in Medicine*. The Research Topic highlights recent findings, innovations, and clinical observations across a diverse range of topics in reproductive and maternal health.

## Reproductive medicine and assisted reproductive technology (ART)

Several papers addressed topics in reproductive medicine, focusing on optimizing protocols in assisted reproductive technologies.

Caradeux et al. review the mechanisms underlying placental dysfunction in pregnancies conceived through oocyte donation. They highlight how immunological factors, absence of corpus luteum, and endometrial preparation protocols may contribute to adverse outcomes, including preeclampsia and fetal growth restriction.

Yan et al. present a randomized controlled trial evaluating dual trigger (GnRH agonist plus hCG) vs. hCG-only for oocyte maturation in normal responders with high immature oocyte rates. Dual trigger significantly improved oocyte maturation, pregnancy, and live birth rates, suggesting it may be a more effective strategy in this population.

Gao et al. conducted a systematic review and meta-analysis to evaluate whether delaying frozen-thawed embryo transfer (FET) improves outcomes after fresh embryo transfer failure or freeze-all cycles. Analyzing over 23,000 cycles, they found no significant differences in live birth, clinical pregnancy, or pregnancy loss rates, suggesting immediate FET is a viable option.

Teng et al. examined the impact of sequential embryo transfer on IVF outcomes in a systematic review and meta-analysis. They found that sequential transfers—especially in women with repeated implantation failure—significantly increased chemical and clinical pregnancy rates compared to conventional methods, though further prospective studies are needed to confirm these benefits.

Li et al. retrospectively evaluated the effects of long-acting GnRH agonist downregulation in ART cycles for patients with adenomyosis. Their findings suggest prolonged downregulation may improve pregnancy outcomes in fresh embryo transfers but not necessarily in frozen cycles. The study highlights the need for individualized downregulation strategies based on treatment type.

## Pregnancy complications

Several studies explored the causes, predictors, and outcomes of various pregnancy complications, aiming to inform better clinical decision-making and management.

Mészáros et al. conducted a meta-analysis to assess the predictive value of the neutrophil-to-lymphocyte ratio (NLR) in the first trimester for preeclampsia. Based on six studies involving 2,469 patients, elevated NLR was associated with later development of preeclampsia. The authors propose NLR as a promising, accessible marker to be incorporated into future first-trimester screening protocols.

Olguín-Ortega et al. examined adverse perinatal outcomes in women with clinical and subclinical histopathological chorioamnionitis. In a historical cohort of 311 cases, they found that both clinical and subclinical forms significantly increased risks for preterm birth, sepsis, and low birth weight, especially in advanced stages and grades. The study emphasizes the diagnostic value of placental histology.

Chen et al. presented a retrospective analysis of fetuses with isolated filar cysts diagnosed by prenatal ultrasound. In most cases, the cysts resolved during pregnancy and all infants were asymptomatic postnatally. The findings suggest that isolated filar cysts are typically benign, and careful ultrasound follow-up can support reassuring counseling.

## Preterm birth and preventive interventions

This section includes studies focused on risk factors for preterm birth and interventions to prevent or manage early delivery.

Park et al. investigated outcomes of repeat cerclage in singleton pregnancies with prolapsed membranes after a prior cerclage. Among 35 women, those with a postoperative cervical length ≥20 mm were significantly more likely to deliver after 26 weeks. The study supports the use of postoperative cervical length as a predictor of cerclage success and gestational prolongation.

Graf et al. analyzed over 1,500 pregnancies with antenatal corticosteroid administration due to imminent preterm birth. The study found that certain risk factors, notably amniotic infection syndrome and placental bleeding, were associated with higher odds of extreme preterm birth. Accurate timing of corticosteroid use remains essential to optimize outcomes in high-risk pregnancies.

Wang et al. explored placental characteristics in monochorionic diamniotic twins with selective fetal growth restriction (sFGR). They found that thick artery–artery anastomoses were more prevalent in late-onset sFGR and associated with delayed onset. These vascular features may modulate the progression of sFGR, suggesting implications for surveillance strategies.

## Surgical and pathological case reports

These contributions offer insights into rare or diagnostically challenging cases that highlight the importance of accurate diagnosis and tailored surgical approaches.

Tan et al. describe a case of struma ovarii misdiagnosed as ovarian cancer. Imaging findings and elevated CA 125 levels initially raised suspicion of malignancy, but pathology revealed thyroid tissue consistent with struma ovarii, alongside Hashimoto's thyroiditis. The case emphasizes diagnostic challenges and the importance of considering this rare entity.

Zhang D. et al. report on a massive retroperitoneal solitary fibrous tumor initially misdiagnosed as an ovarian mass. The tumor's proximity to the external iliac vessels required complex surgical management, including vessel resection and replacement. Histopathology confirmed high-risk solitary fibrous tumor, underscoring the importance of preoperative vascular assessment.

Guo et al. present a case of intravascular leiomyomatosis (IVL) detected via MRI in an asymptomatic woman with presumed benign uterine leiomyoma. The case illustrates the imaging overlap between IVL and common fibroids and stresses the need for surgical excision due to potential for vascular invasion and recurrence.

Zhang M. et al. analyze ultrasonographic features in seven cases of ovarian leiomyoma. The tumors appeared as well-circumscribed hypoechogenic masses, but all were misdiagnosed preoperatively. The report highlights the diagnostic difficulty in distinguishing ovarian leiomyomas from other adnexal tumors and suggests immunohistochemistry as essential for accurate diagnosis.

## Patient perspectives and decision-making

These studies reflect the growing interest in understanding patient preferences and healthcare provider attitudes.

Nguyen et al. conducted a cross-sectional survey on childbirth and pain relief preferences among pregnant women in Vietnam. One-third preferred cesarean delivery, often influenced by social and familial factors. While pharmacological methods were commonly accepted, support from relatives was the preferred non-pharmacological pain relief. The study emphasizes the need for culturally sensitive education and policy interventions.

Lu et al. surveyed 285 healthcare professionals in China to assess attitudes toward uncertain results from prenatal exome sequencing. Most reported limited pre-test counseling about variants of uncertain significance, and uncertain results rarely influenced pregnancy management. The study calls for national guidelines and enhanced training in genetic counseling to improve care in this evolving field.

## Broader trends and research directions

Two articles provide overviews of scientific trends and outcomes associated with maternal health.

Silberstein et al. retrospectively evaluated reproductive outcomes in women who had undergone surgery for ovarian torsion. Among 199 patients, no significant differences in live birth or fertility treatment rates were found before and after the torsion. The findings support that ovarian torsion—when surgically managed—does not adversely impact long-term reproductive potential.

Ye et al. performed a bibliometric and visualized analysis of 2,331 publications on peripartum respiratory complications from 2004 to 2023. They identified major research trends, including venous thromboembolism, COVID-19, and respiratory distress syndromes. The United States led in output and collaboration. Future research may focus on ECMO, cytokine storms, and cardio-respiratory interactions in pregnancy.

In conclusion, this Research Topic captures the breadth and ingenuity of contemporary research in Obstetrics and Gynecology, reflecting both longstanding clinical challenges and emerging frontiers. From innovations in assisted reproduction and improved prediction of pregnancy complications to nuanced patient-centered care and sophisticated imaging diagnostics, the assembled articles illustrate how the field is increasingly defined by precision, personalization, and interdisciplinarity. Notably, contributions addressing AI readiness, genomic uncertainty, and patient decision-making signal a broader shift toward integrative, data-informed, and ethically grounded practices. As Obstetrics and Gynecology evolves, these studies reaffirm the importance of merging foundational clinical insight with cutting-edge research methodologies. Looking forward, the field must continue fostering collaborative inquiry across global contexts to ensure that technological advances translate into equitable, effective, and compassionate care for all individuals across the reproductive lifespan.

